# Sleep interventions in elite sport – a systematic review

**DOI:** 10.17159/2078-516X/2025/v37i1a18811

**Published:** 2025-02-15

**Authors:** SC Bilgoe, S den Hollander, DC Janse van Rensberg, S Hendricks, G Kerkhoffs, V Gouttebarge

**Affiliations:** 1 Amsterdam UMC, location University of Amsterdam, Department of Orthopedic Surgery and Sports Medicine, Meibergdreef 9, Amsterdam, the Netherlands; 2 Amsterdam Collaboration on Health & Safety in Sports (ACHSS), IOC Research Center of Excellence, Amsterdam, the Netherlands; 3 Football Players Worldwide (FIFPRO), Hoofddorp, the Netherlands; 4 Division of Physiological Sciences and Health through Physical Activity, Lifestyle and Sport Research Centre, Department of Human Biology, Faculty of Health Sciences, University of Cape Town, Cape Town, South Africa; 5 Section Sports Medicine, Faculty of Health Sciences, University of Pretoria, Pretoria, South Africa; 6 Carnegie Applied Rugby Research (CARR) Centre, Carnegie School of Sport, Leeds Beckett University, Leeds, UK; 7 Academic Center for Evidence-based Sports Medicine (ACES), Amsterdam, the Netherlands; 8 Amsterdam Movement Sciences, Aging & Vitality, Musculoskeletal Health, Sports, Amsterdam, the Netherlands

**Keywords:** sleep quality, elite athletes, sports medicine

## Abstract

**Background:**

Elite athletes encounter various situations and conditions that may disrupt their sleep, a crucial factor for optimal performance and well-being.

**Objectives:**

The aim of this study was to synthesise existing research on the effect of sleep interventions on sleep quantity and quality in elite sports and to provide evidence-based guidance for athletes, coaches, and other stakeholders in elite sports who seek to enhance sleep quantity and quality.

**Methods:**

This review followed the PRISMA guidelines, whereas the search was executed in September 2023 utilising the electronic databases SCOPUS, PubMed and Web of Science. Studies were included if they met the inclusion criteria.

**Results:**

A total of 1014 studies were retrieved from the databases, and data extraction was performed on 32 studies. The included studies evaluated sleep hygiene education/strategies, acute cold exposure, light therapies, supplementation, neurostimulation/neurofeedback, and other (mindfulness and massage therapy) strategies. Sleep hygiene education was the most effective intervention to improve sleep quantity. Supplementation and light therapy interventions showed improved sleep quality and quantity. Additionally, cold water immersion and mindfulness showed improved sleep quality, but further studies are required for confirmation.

**Conclusion:**

Future research should use reliable and valid methods to improve the quality of evidence and ensure conclusive findings.

Sleep plays an important role in improving performance, promoting health, and ensuring the overall well-being of elite athletes.^1^ Reportedly, most elite athletes do not get the daily recommended hours of sleep per night (7–9 hours),^2^ and, on average, get less sleep than the general population.^3^ Various factors, including training schedules,^4^ frequent travel,^5^ competition demands,^[6,7]^ and caffeine consumption,^8^ have been shown to disrupt the sleep patterns of elite athletes. Sleep disruption can have a significant impact on elite athletes as sleep deficiency has been associated with impaired physical performance,^9^ impaired neurocognitive performance,^10^ and an increased risk for injuries and illnesses.^[11,12]^

Various sleep interventions have been implemented in elite sports to address sleep-related challenges and reduce the risks associated with sleep deprivation. These interventions include napping,^13^ sleep hygiene education,^14^ and postexercise recovery strategies.^15^ Nevertheless, the efficacy and effectiveness of these available interventions in elite sports settings remain uncertain. Some studies have explored the impact of sleep interventions on sleep, recovery and athletic performance.^[16,17]^ However, no study has specifically focused on the effectiveness of sleep interventions on sleep quality and quantity among elite athletes.

This systematic review synthesised the existing research on sleep interventions in elite sports. It addressed the following key research question: What sleep interventions effectively improved the quantity and quality of sleep among elite athletes? The objective was to provide evidence-based guidance for athletes, coaches, and other stakeholders in elite sports who seek to enhance sleep quality and quantity.

## Methods

This review followed the Preferred Reporting Items for Systematic Reviews and Meta-Analyses (PRISMA) guidelines.^18^

### Data sources and search strategy

Three databases [SCOPUS, PubMed and Web of Science (MEDLINE)] were searched for relevant studies in September 2023. The search queries for each database, detailed in Table 1, included ten queries related to sleep interventions in elite or professional sports. No filters were set.

### Eligibility criteria

The eligibility criteria were as follows:

▪ An original research study published in a peer-reviewed journal.
• Studies published in the English language.
• The sample consisted of elite athletes, defined as collegiate, Olympic, or professional athletes (Tiers 3 and 4).^19^
• The study assessed either the quantity or quality of sleep.
• The study analysed the effectiveness of an intervention to improve the quantity and/or quality of sleep.

### Data selection

After duplicate removal, two reviewers (SB and SDH) independently screened all titles and abstracts using the eligibility criteria. Subsequently, all potentially eligible full-text articles were screened. The reference lists of the papers that met the eligibility criteria and the retrieved reviews were searched, and the full texts of any relevant papers were screened for eligibility. At any stage of eligibility, disagreements were resolved by consensus between the two reviewers.

### Data extraction

The following data were extracted and recorded onto a data extraction table: publication details (title, author, year of publication), details of the sample (level of competition, country, sport, age, sex, sample size), methods (study design, intervention, sleep measurements, outcome measures), and results (summary of key findings).

### Methodological quality

The methodological quality of the included studies was assessed using a modified version of a quality assessment scale by Abernethy and Bleakley (Table 2).^20^ Hereby, the quality of the study design, participant descriptions, interventions, outcome measures, assessor blinding, and measurement duration were assessed. Two authors (SB and SDH) independently conducted the assessment and were aware of the study authors, place of publication, results, and they were not blinded to this information. In uncertainty, a third researcher (VG) was consulted to obtain consensus. The included studies could obtain a score of 0, 1 or 2 for each item on the scale, resulting in a maximum possible total score of 16 points (Quality score key detailed in Table 2). The overall quality scores were converted into a percentage value and then rated (0–49%=poor, 50–89%=moderate, and >90%=good). Studies that rated as poor were subsequently excluded.

## Results

### Study selection

**Fig. 1:**
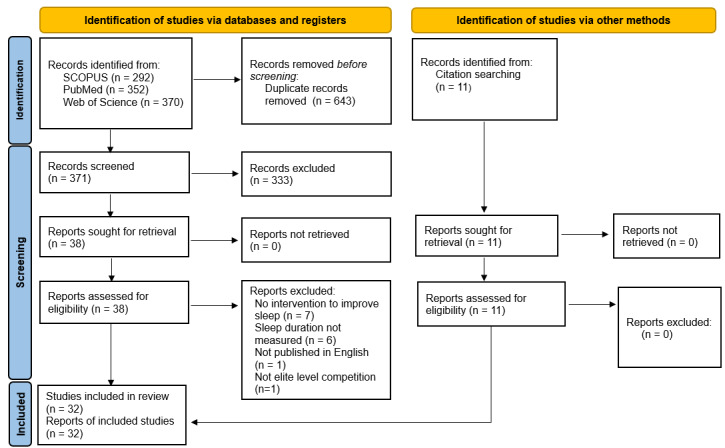
PRISMA 2020 flow diagram of literature search

From 1014 studies retrieved from the databases, 643 duplicates were removed, and a further 333 were excluded (Figure 1). Ultimately, 49 studies were screened for eligibility, resulting in 32 studies for data extraction. Figure 1 represents the detailed flow chart of the study selection.

### Study characteristics

The Table 3 presents detailed information about the reviewed articles. The studies encompassed athletes from 13 countries participating in 16 different sports,^[14,15,21–49]^ with 16 involving only male participants (Table 3).^[14,15,21,24–26,29–35,40,41,49]^ Furthermore, the quality scores of the included studies ranged from 8 to 14, with seven of the studies rated as ‘good’ and 25 as ‘moderate’ (Table 4).

### Sleep interventions

Among the various sleep interventions, sleep hygiene education^[14,21–26]^ and strategy^[27–32]^ followed by supplementation^[40–44]^ were mostly used in the included studies (Table 5). Each study assessed sleep quality and sleep quantity using different measurements. The most common quality assessments were sleep latency, sleep efficiency, wake after sleep onset, and subjective sleep measurements (questionnaires). The most common quantity assessments included time in bed and total sleep time. Seven studies^[14, 21–26]^ examined the effect of sleep hygiene education on several sleep parameters, where improvements in these parameters ranged from 29 % for sleep latency to 75 % for total sleep time. Among sleep hygiene strategies, six studies were included evaluating various strategies such as removal of electronic devices at night,^[27, 28]^ a 10-minute shower at ~40°C before lights out,^29^ sleeping on a high heat capacity mattress,^30^ napping,^31^ and a sleep extension intervention^32^ where improved sleep parameters ranged from 17 % for sleep latency to 40 % for time in bed. Furthermore, five studies^[40–44]^ observed various supplementation interventions where improvements in wake after sleep onset and time in bed reached 33%.

Three studies ^[15,33,34]^ on cold water immersion were included in which sleep latency, sleep efficiency, time in bed, and total sleep time measurements were observed. Two three-day cold water immersion sessions (10 minutes each session) improved sleep latency. In contrast, a combination of 15-minute cold water immersion, compression garments and sleep hygiene recommendations improved time in bed and total sleep time.^[15,33]^

Three studies evaluated the effect of cryostimulation (partial body exposure and whole-body exposure), whereas no improvements in sleep parameters were observed.^[30,35,36]^

As for light therapy, three studies were included that evaluated either bright light therapy or red light therapy, where improved sleep quality ranged from 0% for sleep efficiency to 100% for subjective sleep measurements.^[37–39]^

Three studies evaluated the impact of neurostimulation/neurofeedback on elite athletes during training periods.^[45–47]^ Auditory brainwave entrainment improved subjective sleep measurements, time in bed, and total sleep time.^45^ The other sleep interventions included in our study, an eight-week mindfulness-based stress reduction course and massage therapy, also improved subjective sleep measurements.^48^

## Discussion

Thirty-two studies evaluating various sleep interventions across different elite sports were analysed. The interventions retrieved from the literature included sleep hygiene education and strategies, cold water immersion, cryostimulation, light therapies, supplementation, neurostimulation/neurofeedback, and other (mindfulness-based stress reduction and massage therapy) strategies. Based on the reviewed studies, sleep hygiene education was the most effective intervention to improve sleep quantity. Supplementation and light therapy interventions showed improvements in both sleep quality and quantity. Furthermore, cold water immersion and mindfulness-based stress reduction showed improved sleep quality. However, these findings are based on a limited number of studies; thus, further research is needed to confirm these results.

### Sleep hygiene

Sleep hygiene has been suggested as a potential solution for promoting optimal sleep in athletes, although its effectiveness is limited when implemented as a standalone treatment.^[15,26,50]^ The effects may be influenced by individual differences among athletes, such as their motivation to incorporate sleep hygiene into their long-term routine.^[16,17]^ The reviewed studies indicate that sleep hygiene education in elite athletes can lead to improved sleep quantity.^[14,21–26]^ Our results also indicate the necessity for educating elite athletes about sleep hygiene, enabling them to optimise their sleep patterns effectively. Caia et al.(2018) highlighted the advantages and limitations of a sleep hygiene intervention among professional rugby league athletes.^14^ Implementing a sleep hygiene intervention initially led to notable improvements in time in bed and total sleep time.^14^ However, at the 1-month follow-up, the favourable impacts on time in bed were not sustained, and a decreased sleep efficiency was observed, implying the limited long-term effectiveness of time in bed as a standalone treatment.^14^ The results have conflicted regarding sleep hygiene strategies focused on sleep extension, whether through 40-minute naps or a 3-week sleep extension intervention.^[31,32]^ A 40-minute napping intervention observed no differences in sleep parameters post-intervention. In contrast, a 3-week sleep extension intervention aiming for 10 hours per night showed improved Pittsburgh Sleep Quality Index (PSQI) scores, time in bed and total sleep time in elite athletes.^[31,32]^ Another strategy, namely removing electronic devices, reported no improvements in sleep parameters.^[27,28]^ Furthermore, a study evaluating showering at ~40°C before bed and another study evaluating the use of high-heat capacity mattresses reported positive sleep results in elite athletes.^[29,30]^ The ability to assess and compare the effectiveness of sleep hygiene is limited, as these studies evaluated different strategies across various sports. Hence, it remains difficult to conclude which sleep hygiene strategies are most effective as this effect may differ across sport types due to contextual and individual differences.

### Cold exposure

Elite athletes increasingly use acute cold exposure as a recovery strategy, whether through cold water immersion or cryostimulation.^[51,52]^ Our reviewed articles included two types of cryotherapy: whole-body, where the entire body is exposed, and partial-body, where specific areas are targeted, excluding the head.^36^ Partial-body cryostimulation at −180°C for 180 seconds among professional soccer players showed significant reductions in the number of movements during the night post-intervention.^35^ Furthermore, whole-body cryostimulation among elite rugby athletes and synchronised swimmers did not show differences in sleep parameters post-intervention.^[30,36]^ The existing literature regarding cryostimulation is still limited, and it shows varied results on sleep quality among elite athletes. Therefore, additional research is necessary to understand the effectiveness before recommending the implementation of cryostimulation as a strategy to promote sleep. Another cold exposure intervention we reviewed in our study was cold water immersion,^[15,33,34]^ where Robey et al. (2013) did not observe any effects on sleep after 15-minute sessions among elite cyclists and triathletes postexercise.^34^ However, Lastella et al.(2019) observed shorter sleep latency in the intervention condition compared to the control condition.^33^ Furthermore, 15-minute cold water immersion combined with other strategies such as three hours of wearing full-body compression garments and sleep hygiene recommendations (incl. establishing a cool temperature (19±2°C) and low-light environment, avoiding the use of electronic devices and excessive light 30 minutes before 9.30pm) after daily on-court tennis training and matchplay sessions resulted in improvements in sleep quantity.^15^

### Light therapy

Existing literature suggests that artificial light exposure can shift the circadian rhythm, playing a crucial role in our daily hormonal and behavioural patterns of melatonin secretion, sleepiness, alertness, and performance.^53^ Bright light therapy may impact melatonin suppression, affect the body clock, and increase evening alertness when it usually starts to drop.^54^ Rosa et al.(2018) reported a delayed sleep/wake cycle after bright light therapy in the evening. Thompson et al.(2012) reported that bright light therapy did not substantially reduce jet lag symptoms in athletes after travelling.^[37,38]^ As for red light therapy, which does not impact melatonin suppression, Zhao et al.(2012) reported increased PSQI scores following red light treatment in the evening.^[39,55]^ These results should be interpreted cautiously as the assessment tool (PSQI) is not validated in an athletic population and may be unreliable in examining sleep quality over a short-term period.^[56,57]^

### Supplementation

Our findings indicate that probiotic supplementation may improve objective and subjective sleep parameters among professional soccer and rugby athletes.^[40,41]^ These results should be interpreted cautiously as these two studies also contain limitations. In the study among professional rugby players, an additional probiotic was used to prevent diarrhoea, possibly influencing the outcome.^40^ Moreover, sleep among soccer players who received probiotic strains for 30 days was measured using actigraphy.^41^ However, this method may not be sensitive enough to detect changes in sleep latency in players without sleep problems, potentially leading to underreported sleep latency values.^58^ Nonetheless, probiotic supplementation has been shown to improve subjective sleep quality in non-athlete populations.^59^ Recent literature suggests that probiotics may improve athletes’ sleep by reducing muscle soreness and influencing the sleep/wake cycle through melatonin synthesis.^[40,60]^ While recent studies showed promising results regarding the relationship between probiotics and athletes’ sleep, future research that uses more thorough methods for assessing sleep (e.g., polysomnography) and implements stricter control of confounding variables is necessary to accurately evaluate the true effect of probiotic supplementation on athletes’ sleep.

Other nutritional strategies have positively affected sleep indices in an elite athlete population, including kiwifruit and tart cherry juice consumption.^[43,44]^ Kiwifruit consumption for four weeks improved PSQI scores among elite sailing and track and field athletes.^43^ Additionally, tart cherry juice intake among female hockey players improved time in bed, sleep efficiency and wake after sleep onset.^44^ Both of these nutrient supplementation strategies have shown positive effects on sleep indices in the general population due to their level of melatonin and serotonin.^[61–63]^ Furthermore, kiwifruit contains a substantial amount of folate, potentially improving sleep quality, as a folate deficiency is associated with insomnia.^[62,64]^ However, most studies exploring the use of tart cherry juice in athletic populations have focused on its impact on various aspects of recovery, such as muscle soreness,^[61,65,66]^ thus emphasising the need for future studies focusing on its impact on sleep parameters among elite athletes. Further on the nutrient supplementation evaluation, consuming pre-sleep α-lactalbumin in a semi-professional female rugby team during a competition season led to improved sleep latency.^42^ In recent literature, α-lactalbumin has been identified as containing the highest levels of tryptophan, an amino acid serving as a precursor to the sleep-promoting hormones melatonin and serotonin.^[67,68]^

### Neurostimulation/neurofeedback

The electroencephalogram cortical oscillations can be categorised into specific frequencies linked to different states of brain functioning (delta [1.5–6 Hz], theta [6.5–8 Hz], alpha [8.5–12 Hz], beta [8.5–30 Hz]).^69^ Brainwave entrainment requires stimulating the brain during sleep with frequencies between 1 and 9 Hz, supporting the healthy human sleep cycle.^[70,71]^ Abeln et al.(2013) reported improvements in subjective sleep quality after eight weeks of auditory brainwave entrainment among high-level soccer players.^45^ Furthermore, eyes-open alpha training among elite gymnasts and mental coaching in combination with alpha power feedback among elite athletes did not significantly improve sleep parameters.^[46,47]^

### Other

The other strategies we evaluated, specifically eight weeks of mindfulness-based stress reduction for elite rowing athletes and massage therapy for elite para-cyclists, both enhanced subjective sleep parameters.

### Limitations

While our systematic review suggests that many of the interventions positively affect sleep, these findings are based on a limited number of studies. Furthermore, most of the studies presented a substantial risk of bias, also mentioned in a recent consensus statement on sleep among athletes.^57^ The statement emphasises the necessity for more consistent, reliable, and valid research methods in studies on athlete’s sleep, given the poor quality of current research on this topic.^57^ Moreover, each study assessed sleep quality and quantity using different outcome measures. The variability in the assessment of sleep quality and sleep quantity between the studies made it difficult to synthesise and compare the results.

### Recommendations

#### Recommendations for practice

Athletes, coaches, and other stakeholders involved in elite sports should receive sleep education to raise awareness about the importance of sleep and offer insights into different strategies that can be implemented. Sleep education should cover information on the appropriate night-time sleep quantity, daytime sleep quantity (naps), good sleep hygiene, and the potential impact of late-night or early-morning training on sleep health as recommended in the recent consensus statement.^57^ Additionally, practitioners should regularly screen athletes for sleep problems and refer them to a sleep specialist for clinical diagnosis. As a result, the proper treatment can be provided, as some sleep interventions may be ineffective in the presence of a sleeping disorder. Notably, caution is necessary when using sleep monitors, as athletes can become concerned about the data, leading to reduced sleep quality due to increased anxiety.^57^

#### Recommendations for researchers

Empirical studies regarding sleep health among elite athletes are still limited. Therefore, future researchers and clinicians should collaborate with elite athletes, teams, and organisations across different sports types to increase the quantity and quality of current studies in this area. To improve the quality of these studies, researchers should design longitudinal studies to assess the long-term effectiveness of sleep interventions by using validated measurement tools to objectively and subjectively assess sleep parameters. Additionally, researchers should investigate how sport-specific factors, individual differences (e.g., sex, age, race/ethnicity, and chronotype), and other factors (e.g., training, travel, and competition schedules) can influence the effectiveness of sleep interventions. Furthermore, sleep measurements show major heterogeneity among studies in elite athletes, highlighting the need to develop standardised guidelines for measuring sleep parameters in this population. This review’s findings should also contribute to future research to identify and develop effective evidence-based sleep interventions for elite athletes.

## Conclusion

Our systematic review suggests that sleep hygiene education improves sleep quantity in elite athletes. The remaining strategies, such as supplementation, light therapy, mindfulness-based stress reduction, and cold water immersion, positively affect elite athletes’ sleep health. Still, more studies are needed to confirm these results. The variability in the assessment of sleep quality and sleep quantity between the studies made it difficult to synthesise and compare the results. Future research should implement consistent, reliable, and valid methods, thereby enhancing the quality of evidence and allowing for more conclusive findings.

## Figures and Tables

**Table 1:** Search Queries

Query	PubMed	Web of Science (MEDLINE)	Scopus
**1**	Sleep[Title/Abstract] AND Intervention[Title/Abstract] AND Elite[Title/Abstract] AND Sports[MeSH Terms]	AB=(Sleep) AND AB=(intervention) AND AB=(elite) AND MHX=(sports)	TITLE-ABS-KEY (sleep AND intervention AND elite AND sport)
**2**	Sleep[Title/Abstract] AND Education[Title/Abstract] AND Elite[Title/Abstract] AND Sports[MeSH Terms]	AB=(Sleep) AND AB=(education) AND AB=(elite) AND MHX=(sports)	TITLE-ABS-KEY (sleep AND education AND elite AND sport)
**3**	Sleep[Title/Abstract] AND nap*[Title/Abstract] AND Elite[Title/Abstract] AND Sports[MeSH Terms]	AB=(Sleep) AND AB=(nap*) AND AB=(elite) AND MHX=(sports)	TITLE-ABS-KEY (sleep AND nap* AND elite AND sport)
**4**	Sleep[Title/Abstract] AND Recovery[Title/Abstract] AND Elite[Title/Abstract] AND Sports[MeSH Terms]	AB=(Sleep) AND AB=(recovery) AND AB=(elite) AND MHX=(sports)	TITLE-ABS-KEY (sleep AND recovery AND elite AND sport)
**5**	Sleep[Title/Abstract] AND Hygiene[Title/Abstract] AND Elite[Title/Abstract] AND Sports[MeSH Terms]	AB=(Sleep) AND AB=(hygiene) AND AB=(elite) AND MHX=(sports)	TITLE-ABS-KEY (sleep AND hygiene AND elite AND sport)
**6**	Sleep[Title/Abstract] AND Intervention[Title/Abstract] AND Professional[Title/Abstract] AND Sports[MeSH Terms]	AB=(Sleep) AND AB=(intervention) AND AB=(Professional) AND MHX=(sports)	TITLE-ABS-KEY (sleep AND intervention AND Professional AND sport)
**7**	Sleep[Title/Abstract] AND Education[Title/Abstract] AND Professional[Title/Abstract] AND Sports[MeSH Terms]	AB=(Sleep) AND AB=(education) AND AB=(Professional) AND MHX=(sports)	TITLE-ABS-KEY (sleep AND education AND Professional AND sport)
**8**	Sleep[Title/Abstract] AND nap*[Title/Abstract] AND Professional[Title/Abstract] AND Sports[MeSH Terms]	AB=(Sleep) AND AB=(nap*) AND AB=(Professional) AND MHX=(sports)	TITLE-ABS-KEY (sleep AND nap* AND Professional AND sport)
**9**	Sleep[Title/Abstract] AND Recovery[Title/Abstract] AND Professional[Title/Abstract] AND Sports[MeSH Terms]	AB=(Sleep) AND AB=(recovery) AND AB=(Professional) AND MHX=(sports)	TITLE-ABS-KEY (sleep AND recovery AND Professional AND sport)
**10**	Sleep[Title/Abstract] AND Hygiene[Title/Abstract] AND Elite[Title/Abstract] AND Sports[MeSH Terms]	AB=(Sleep) AND AB=(hygiene) AND AB=(elite) AND MHX=(sports)	TITLE-ABS-KEY (sleep AND hygiene AND elite AND sport)

**Table 2:** Study quality score key

Study reference:	Score

**1. What study design was used and, where relevant, how were participants allocated?**	
2 = mixed design, randomized and counterbalanced between conditions	
1 = repeated measures/within subjects only OR mixed design but not randomized.	
0 = between groups only	

**2. Was the assigned intervention concealed before allocation?**	
2 = adequate	
1 = unclear	
0 = inadequate/impossible	

**3. Were the outcome assessors blinded to treatment status?**	
2 = effective action taken to blind assessors	
1 = small or moderate chance of unblinding of assessors	
0 = not mentioned or not possible	

**4. Were the inclusion and exclusion criteria (age, previous injury, sport) clearly defined?**	
2 = clearly defined	
1 = inadequately defined	
0 = not defined	

**5. Were the intervention and control group comparable at entry?**	
2 = good comparability of groups, or confounding adjusted for in analysis/within group design only	
1 = confounding small; mentioned but not adjusted for	
0 = large potential for confounding, or not discussed	

**6. Were the interventions clearly defined?**	
2 = clearly defined interventions were applied	
1 = clearly defined interventions were applied but the application was not standardized	
0 = intervention and/or application were poorly or not defined	

**7. Were the outcome measures used clearly defined?**	
2 = clearly defined	
1 = adequately defined/recorded	
0 = not adequately defined/recorded	

**8. Was the follow-up period sufficient to appropriately measure the effects of the intervention?**	
2 = active surveillance and appropriate duration	
1 = active surveillance, but inadequate duration	
0 = surveillance not active or not defined	

Total score (16=100%)	

**Table 3:** Study characteristics of reviewed article

Study	Population				Methods			Results
	Country	Age years (mean±SD, or range)	N (% male)	(Sport/athletes)	Intervention	Design	Sleep Measurements	
**Sleep hygiene education**								
**Caia et al.** ^14^	Australia	25.4±3.3	24 (100)	Rugby	Two Sleep hygiene education seminars or no sleep hygiene education.	Case study	Wrist actigraphy (BT, SOL, WASO, TIB, SD, SE)	The first sleep hygiene education resulted in an earlier bedtime (ES=0.53±0.48), more TIB (ES=0.53±0.49), and increased sleep duration (ES=0.47±0.44). A second sleep hygiene education resulted in more TIB (ES=0.84±0.50) but reduced sleep efficiency (ES=1.15±0.48).
**Driller et al.** ^21^	New Zealand	23±4	9 (100)	Cricket	Sleep hygiene education	ED	Wrist actigraphy (TST, TTB, SE, SL, WASO, WE, WED, SOT, WT, SOV, WV), ASBQ, ESS, PSQI questionnaires	Post intervention showed significant improvements in the ESS (−0.51, p=0.01) and PSQI (−0.71, p<0.01) questionnaires, sleep efficiency (1.38, p<0.01) and SOV (−0.47, p=0.04).
**Dunican et al.** ^22^	Australia	(Players) 25±2, (Coaches) 42±15	17 (18)	Basketball	A sleep-education program (individual consultations and team education)	ED	Wrist actigraphy (alertness, SOL, SOT, SD, WASO, WT, SE, TIB), ISI, ESS, OSA questionnaires	No significant differences were observed post-intervention among the players. Among the coaches there was a significant increase in SD (with 49 minutes, p<0.01), an earlier SOT (by 19 minutes, p=0.04), an increase in TIB (50 minutes, p<0.01), and a later wake-up time each morning (by 31 minutes, p=0.01) post intervention.
**O’Donnell et al.** ^23^	New Zealand	23 ± 6	26 (0)	Netball	Sleep-hygiene education	Single group pre-post design	Wrist actigraphy (TST, SE, TTIB, SL, WE, WV, SOT, WT)	A significant improvement was observed in TST (ES:0.39, p=0.01), WV (ES: −0.70, p=0.03) and WED (ES: −0.57, p=0.03) post intervention. No significant differences were found for SE, SL, TTB, WE, SOV, SOT and WT post intervention.
**Sargent et al.** ^24^	Australia	23.5±3.7	19 (100)	Cricket	Provision of sleep targets and feedback on compliance (intervention) versus only feedback on compliance (Control)	RCT	Wrist actigraphy (SOL, SO, SE), Sleep diary (Sleep-wake behavior), ESS, Morningness - Eveningness Questionnaire	There was an increased average cumulative sleep duration (+36 minutes, p=0.039), decreased cumulative time in bed (+42 minutes, p=0.040) and increased time in bed (+36 minutes, p=0.016) in week 2 compared to week 1 among the athletes in the intervention group.
**Tuomilehtoet al.** ^25^	Finland	17–40	40 (100)	Ice hockey	General sleep counselling and individual treatment plan (Intervention) versus general sleep counselling (Control)	Exploratory observational 1-year follow-up study	PSG (Sleep recording time, TST, TIB, SL), BNSQ Questionnaire	Higher points were scored in self-assessed sleep quality between the first- and second-night’s sleep (5.97±4.18, p<0.0005) after Individual treatment and General sleep counselling.
**Van Ryswyk et al.** ^26^	Australia	23.7±2.0	25 (100)	Football	Six-week sleep optimisation programme	Prospective intervention study	Wrist actigraphy (SD, SE, SOL, WASO), Sleep diary (TIB, daytime naps, intake of caffeine and alcohol), ESS, PSQI Questionnaire	An increase in self-reported TST (+20 minutes, p<0.05) and sleep efficiency (+2%, p< 0.05) were observed post intervention.
**Sleep hygiene strategies**								
**Jones et al.** ^27^	Australia	17±1	Water polo 26 (46) Triathlon 23 (65)	Water polo, Triathlon	Removal of electronic devices	RCT	Wrist actigraphy (SD, SE, SOL, WASO, TIB), PSQI, ESS, ISI Questionnaires, sleep diary (SO, Sleep quality)	The water polo athletes who received the intervention went to bed earlier than the control group on the first night. Among the Triathlon athletes there was no difference between the intervention and control group regarding sleep quantity.
**Dunican et al.** ^28^	Australia	17.2±5.1	18 (56)	Judo	Removal of electronic devices	Observational design	Wrist actigraphy (SD, SE, WASO, SOT, SL, WT), sleep diary (SD, WT), sleep-related questionnaires (BQS, ESS, ISI)	No significant differences were observed.
**Whitworth-Turner et al.** ^29^	England	18±1	11 (100)	Soccer	10-min showering at ~40°C before lights out	RXO	Wrist actigraphy (TOLO, TST, SOL, WASO, TOFA, SE), Sleep diary (SOL, Recall of awakenings, SQ)	A decrease in sleep latency (−7 minutes, p<0.01) and an increase in sleep efficiency (+2%, p<0.01) was observed in the shower condition.
**Aloulou et al.** ^30^	France	20.8±1.0	19 (100)	Rugby	MAT and/or 3 minutes of WBC, 2 hours before bedtime and/or CONT	RXO	PSG (SOL, WASO, TST)	Lower WASO (β=−10.5 min, p<0.01) and higher REM (β =+2.8%, p<0.05) for MAT compared with CONT. WBC showed no effect on sleep architecture.
**Souabni et al.** ^31^	France	27.6±4.7	10 (100)	Basketball	40-minute napping	RXO	Wrist actigraphy (TST, TIB, SE, SOL, WASO), Sleep diary (VAS-scale)	No significant differences between CON and NAP conditions were observed in TIB, TST, SE, WASO, and VAS.
**Swinbourne et al.** ^32^	New Zealand	25±2.7	25 (100)	Rugby	3-week Sleep extension intervention (10 hours of sleep per night)	Pre-post control-trial intervention study design	Wrist actigraphy (TTIB, SL, SD. TST, SE, WASO), PSQI, ESS questionnaires, Sleep diary	Moderate improvements were observed in PSQI scores (−24.8±54.1%), TST (6.3±6.3%) and TIB (7.3±3.6%) post-intervention.
**Cold water immersion**								
**Lastella et al.** ^33^	Australia	21.1±1.7	10 (100)	Cycling	10-minute sessions of CWI and Placebo recovery session (ultrasound turned off)	RXO	Wrist actigraphy (SL, TIB, SE), sleep diary (Bedtime, Getup time, subjective sleep quality)	Between cold water immersion and placebo conditions there were no observed differences in sleep/wake behaviours except for sleep latency, which was shorter in the CWI condition. (ES: −0.24, p=0.03)
**Robey et al.** ^34^	Australia	26.0±4.4	11 (100)	Cycling and Triathletes	Post exercise cold water immersion (4 weeks)	RCT	Wrist actigraphy (TST, SE, SOL, SL, WASO, TIB), Saliva samples	No differences were observed between conditions for TST, SE, SOL, REM onset latency, and WASO.
**Duffield et al.** ^15^	Australia	20.9±3.6	8 (100)	Tennis	Cold water immersion, Compression garments and sleep hygiene recommendations	RXO	Wrist actigraphy (SD, SE, SL, TIB, TST)	Improvements in TIB, and actual sleep time (p>0.05, d=2.41) were observed post-intervention.
**Cryostimulation**								
**Aloulou et al.** ^30^	France	20.8±1.0	19 (100)	Rugby	MAT and/or 3 minutes of WBC, 2 hours before bedtime and/or CONT	RXO	PSG (SOL, WASO, TST)	WBC showed no effect on sleep architecture.
**Douzi et al.** ^35^	France	24.8±5.5	9 (100)	Soccer	Cryostimulation with different exposure duration at 180°C, (180-s exposure, twice 90-s exposure) or no cryostimulation	ED	Wrist actigraphy (SE, TST, Wake time), SSQP questionnaire (subjective sleep quality)	Partial-body cryostimulation at 180°C for 180-s showed a significant reduction in number of movements during the night (g> −1.24, p values ranged from 0.01 to 0.05) compared to the other conditions. A non-significant improvement was observed in sleep efficiency after the 180-s exposure (p<0.09).
**Schaal et al.** ^36^	France	20.4±0.4	10 (0)	Synchronized Swimming	2 week period WBC	RXO	Wrist actigraphy (TTIB, Sleep start – bedtime, get-up time, SE, Actual sleep time)	No significant improvements were observed after WBC.
**Light therapies**								
**Rosa et al.** ^37^	Brazil	24.8±3.4	22 (50)	Swimming	5-day Bright light therapy	WI-RM	Wrist actigraphy (TST, SL, SE, WASO), Sleep diary (Bedtime, wake-up time)	Later bedtimes (F1,21=7.30, p<0.01, ES=0.25) and an increase in TST (F1,21=2.9, p=0.01, ES=0.12) were observed post-intervention.
**Thompson et al.** ^38^	Portugal	26±4	20 (0)	Soccer	45–60 min of Bright light therapy for 2 days	Parallel-group randomised controlled trial	VAS-score (SL, sleep quality, start-time, waking-time)	Overall ratings of sleep showed a small improvement after the first night in the intervention group compared to the control group.
**Zhao et al.** ^39^	China	18.60±3.60	20 (0)	Basketball	Red-light therapy for 30 minutes at night. (14 days)	Randomised parallel pretest-posttest design	PSQI questionnaire, Serum melatonin	Improvements in the intervention group were observed in PSQI scores (T18= −4.55, p<0.001), SD (F1,18=5.36, p=0.03) and sleep latency (F1,18=5.65, p=0.03).
**Supplementation**								
**Harnett et al.** ^40^	Australia	27.0±3.2	29 (100)	Rugby	17-week Probiotic supplementation or placebo	RCT	Subjective sleep ratings (sleep quantity and quality), saliva samples (melatonin levels), enzymeimmuno-assay kits (muscle soreness and leg heaviness scores)	As muscle soreness and leg heaviness scores decreased in the probiotic group, sleep quantity and quality scores increased.
**Quero et al.** ^41^	Spain	(Sedentary individuals) 23.04±2.09, (Soccer) 20.66±1.39	27 (100)	Soccer, sedentary individuals	Synbiotic Gasteel Plus® supplementation or placebo	Triple-blinded, randomised, placebo-controlled study	Wrist actigraphy (SL, SE), blood and saliva samples, (catecholamines and stress-hormones)	Significant improvements were observed in sleep efficiency (%) (from 87.46±6.09 to 90.8±3.17) and sleep latency (from 1.38±0.97 to 0.88±0.74) post-intervention.
**Gratwicke et al.** ^42^	Australia	23.8±5.2	16 (0)	Rugby	α-lactalbumin supplement or placebo	RCT	Wrist actigraphy (TST, SE, SOL, WASO), sleep diary (bedtime, wake time).	Participants in the a-LAC group had a reduced SOL during the bye (from 23.3±16.3 to 11.6±13.4 min) and away game (from 23.3±16.3 to 17.0±11.5 min) compared to the baseline. No differences were observed in the PLA group compared to baseline.
**Doherty et al.** ^43^	Ireland	(sailing) 24.56±4, (athletics) 21.17±2.93	15 (60)	Sailing, athletics	Four-week kiwi fruit consumption	Open-label trial	RESTQ sport, PSQI, CSD-C, RU-SATED questionnaires, sleep diary (SOL, WASO, TIB, TST, SE, Fatigue, sleep quality)	Clinically significant improvements were observed in PSQI scores post-intervention. (mean difference: 2.5, p=0.002). There were non-significant improvements in SOL (mean difference:1, p=0.18), SD (mean difference:1, p=0.15) and sleep efficiency (mean difference:1.5, p=0.09) post-intervention.
**Chung et al.** ^44^	Germany	21.30±1.06	19 (0)	Hockey	48-hour period of TCJ or placebo intake	RCT	ASBQ, ASSQ questionnaires (sleep quality levels), wrist actigraphy (SE, TTB, TST, WASO, NOA, AAL, MI, FI), and blood samples (melatonin and cortisol levels)	Short-term intake of TCJ showed improvements in TTB (ES=0.31) and WASO (ES=0.22) in female hockey players.
**Neurostimulation/neurofeedback**								
**Abeln et al.** ^45^	Germany	16.3± 91.02	39 (74)	Soccer	Eight weeks auditory brainwave entrainment	Mixed-methods	Sleep diary, SSA (subjective sleepiness), psychophysical state of participants (Mood)	Eight weeks of auditory stimulation showed significant improvement in subjective sleep quality (x^2^=23.57, p=0.01)
**Dekker et al.** ^46^	Netherlands	22.0±2.3	12 (67)	Gymnast	Alpha power training or random beta power training	RCT	EEG measurements (alpha power), PSQI questionnaire	A non-significant decrease in sleep complaints after Alpha power training compared to random beta training were observed in the PSQI questionnaire (Group × Effect Measurement interaction: F(1,12) = 3.33, p=0.09)
**Rijken et al.** ^47^	Netherlands	(Soccer) 21–32, (16–38)	Soccer 11 (100), Track and field 10 (25)	Soccer, Track and field	(Soccer) Mental coaching + HRV Biofeedback, (Track and field) Mental coaching + Neurofeedback	Prospective cohort study design	EEG (Alpha power), PSQI questionnaire	No significant differences were observed in sleep parameters.
**Other**								
**Jones et al.** ^48^	United States of America	18–23	27 (0)	Rowing	8-week Mindfullnes-based stress reduction course with a regular athletic training program (MBSR) or an athletic training program alone. (control)	Quasi-randomised control trial	PSQI, ESS questionnaire, wrist actigraphy (TST, WASO, SE)	Improvements in the ESS score, ([t = 2.957, p = 0.010, Cohen’s d = 0.763, 95% CI (0.861, 5.406) and sleep efficiency [t = −2.787, p = 0.024, Cohen’s d = 0.929, 95% CI (−3.155, −0.298)] were observed post-intervention in the MBSR group.
**Kennedy et al.** ^49^	United States of America	39.14±9.23	9 (100)	Para-cycling	Massage therapy	A quasi-experimental, convergent, parallel, mixed-methods design	SF-36 or SF-36V, MT session intake and exit questionnaires (stress, sleep, physical functioning)	Significant improvements were observed in subjective sleep measurements among the athletes. (from 4.1±1.2 at baseline to 2.5±1.5 at final visit).

AAL, average awakening length; BT, bedtime; CBT, core body temperature; CONT, control; CSD-C, Consensus Sleep Diary-Core; CWI, cold water immersion; ED, experimental design; ES effect size; FI, fragmentation index; MAT, high-heat capacity mattress; MI, movement index; NOA, number of awakenings; PSG, polysomnography recordings; PSG, polysomnography; PSQI, Pittsburgh Sleep Quality Index; RCT, randomized controlled trial; RESTQ, Recovery Stress Questionnaire for athletes: RU-SATED, Regulatory, Satisfaction, Alertness, Timing, Efficiency and Duration; RXO, randomized cross-over design; SD, sleep duration; SE, sleep efficiency; SF-36, The MOS 36-item short-form health survey; SF-36V, physical functioning scale for use with veterans; SL, sleep latency; SOL, sleep onset latency; SOT, sleep onset time; SSA, Self-Assessment questionnaire of Sleep and Awakening quality; TCJ, tart cherry juice; TIB, time in bed; TOFA, time of final awakening; TOLO, time of lights out; TST, total sleep time; TTB, total time in bed; VAS, visual analogue scale; WASO, wake after sleep onset; WBC, whole-body cryotherapy session: WE, wake episodes per night; WED, wake episode duration; WI-RM, within subjects repeated measures; WT, wake time; WV, wake variance

**Table 4:** Quality scores of reviewed articles

Study	Q1	Q2	Q3	Q4	Q5	Q6	Q7	Q8	Total score	Quality rating
Abeln et al.^45^	1	0	0	2	2	2	2	1	10	M
Aloulou et al.^30^	1	2	0	1	2	2	2	1	11	M
Beaven et al.^72^	2	0	0	0	2	2	2	2	10	M
Caia et al.^14^	1	0	0	0	2	2	2	2	9	M
Chung et al.^44^	2	2	2	2	2	2	2	1	14	G
Dekker et al.^46^	2	2	2	0	2	2	2	2	14	G
Doherty et al.^43^	1	0	0	0	2	2	2	2	8	M
Douzi et al.^35^	2	0	0	1	2	2	2	2	11	M
Driller et al.^21^	1	0	0	2	2	2	2	1	10	M
Duffield et al.^15^	1	0	0	0	2	2	2	2	9	M
Dunican et al.^22^	1	0	0	0	2	2	2	0	7	M
Dunican et al.^28^	1	0	0	0	1	2	2	2	8	M
Gratwicke et al.^42^	2	2	2	2	2	2	2	1	15	G
Harnett et al.^40^	2	2	2	0	2	2	2	2	14	G
Jones et al.^48^	2	0	0	2	2	2	2	1	11	M
Jones et al.^27^	2	0	0	0	2	2	2	1	9	M
Kennedy et al.^49^	1	0	0	2	2	2	2	1	10	M
Lastella et al.^33^	2	0	0	1	2	2	2	1	10	M
O’Donnell et al.^23^	1	0	0	0	2	2	2	1	8	M
Quero et al.^41^	2	2	2	2	2	2	2	1	15	G
Rijken et al.^47^	0	0	0	1	2	2	2	2	9	M
Robey et al.^34^	2	0	0	0	2	2	2	2	10	M
Rosa et al.^37^	1	0	0	2	2	2	2	2	11	M
Sargent et al.^24^	2	0	0	2	2	2	2	1	11	M
Schaal et al.^36^	2	0	0	2	2	2	2	2	12	M
Souabni et al.^31^	2	0	0	2	2	2	2	2	12	M
Swinbourne et al.^32^	1	0	0	1	2	2	2	1	9	M
Thompson et al.^38^	2	2	2	1	2	2	2	1	14	G
Tuomilehto et al.^25^	1	2	0	0	0	2	2	2	8	M
Van Ryswyk et al.^26^	1	0	0	1	2	2	2	2	10	M
Whitworth-Turner et al.^29^	2	0	0	1	2	2	2	2	11	M
Zhao et al.^39^	2	2	0	2	2	2	2	2	14	G

M, moderate; G, good

**Table 5:** Measurements used to assess sleep quality and quantity

Intervention	Improved sleep quality % (n)				Improved sleep quantity % (n)	
	SL	SE	WASO	Subjective sleep measurements (Questionnaires)	TIB	TST
Hygiene education (n=7)	29 (2)	43 (3)	20 (1)	20 (1)	29 (2)	75 (6)
Hygiene strategy (n=6)	17 (1)	20 (1)	33 (2)	33 (1)	40 (2)	33(2)
Cold water immersion (n=3)	33 (1)	0 (0)	0 (0)	0 (0)	33 (1)	33 (1)
Cryostimulation (n=3)	0 (0)	0 (0)	0 (0)	0 (0)	0 (0)	0 (0)
Light therapy (n=3)	33 (1)	0 (0)	0 (0)	100 (2)	50 (1)	67 (2)
Supplementation (n=5)	67 (2)	25 (1)	33 (1)	50 (2)	33 (1)	25 (1)
Neurostimulation/neurofeedback (n=3)	0 (0)	0 (0)	0 (0)	33 (1)	100 (1)	100 (1)
Other (MBSR and massage therapy) (n=2)	0 (0)	100 (1)	100 (1)	67 (2)	0 (0)	0 (0)
Hygiene education (n=7)	29 (2)	43 (3)	20 (1)	20 (1)	29 (2)	75 (6)

MBSR, Mindfulness-based stress reduction course; n, number of studies; SE, sleep efficiency; SL, sleep latency; TIB, time in bed; TST, total sleep time; WASO, wake after sleep onset.
